# Searching New Solutions for NiTi Sensors through Indirect Additive Manufacturing

**DOI:** 10.3390/ma15145007

**Published:** 2022-07-19

**Authors:** Pedro Carreira, Daniel Gatões, Nuno Alves, Ana Sofia Ramos, Maria Teresa Vieira

**Affiliations:** 1 CDRSP—Centre for Rapid and Sustainable Product Development, Polytechnic Institute of Leiria, 2411-901 Leiria, Portugal; pedrosilvacarreira@gmail.com (P.C.); nuno.alves@ipleiria.pt (N.A.); 2 University of Coimbra, CEMMPRE—Centre for Mechanical Engineering, Materials and Processes, Department of Mechanical Engineering, Polo II, R. Luís Reis Santos, 3030-788 Coimbra, Portugal; daniel.gatoes@uc.pt (D.G.); teresa.vieira@dem.uc.pt (M.T.V.)

**Keywords:** Nickel-Titanium (NiTi), Shape Memory Alloys (SMAs), Metal Extrusion (MEX), additive manufacturing (AM), Titanium Hydride (TiH_2_)

## Abstract

Shape Memory Alloys (SMAs) can play an essential role in developing novel active sensors for self-healing, including aeronautical systems. However, the NiTi SMAs available in the market are almost limited to wires, small sheets, and coatings. This restriction is mainly due to the difficulty in processing NiTi through conventional processes. Thus, the objective of this study is to evaluate the potential of one of the most promising routes for NiTi additive manufacturing—material extrusion (MEX). Optimizing the different steps during processing is mandatory to avoid brittle secondary phases formation, such as Ni_3_Ti. The prime NiTi powder is prealloyed, but it also contains NiTi_2_ and Ni as secondary phases. The present study highlights the role of Ni and NiTi_2_, with the later having a melting temperature (Tm = 984 °C) lower than the NiTi sintering temperature, thus allowing a welcome liquid phase sintering (LPS). Nevertheless, the reaction of the liquid phase with the Ni phase could contribute to the formation of brittle intermetallic compounds, particularly around NiTi and NiTi_2_ phases, affecting the final structural properties of the 3D object. The addition of TiH_2_ to the virgin prealloyed NiTi powder was also studied and revealed the non-formation of Ni_3_Ti for a specific composition. The balancing addition of extra Ni revealed priority in the Ni_3_Ti appearance, emphasizing the role of Ni. Feedstocks extruded (filaments) and green strands (layers), before and after debinding & sintering, were used as homothetic of 3D objects for evaluation of defects (microtomography), microstructures, and mechanical properties. The composition of prealloyed powder with 5 wt.% TiH_2_ addition after sintering showed a homogeneous matrix with the NiTi_2_ second phase uniformly dispersed.

## 1. Introduction

NiTi is classified as a shape memory alloy (SMA), and is defined as an intermetallic material, with the ability to restore its previously defined shape when exposed to a specific thermal cycle, either through shape memory effect or superelasticity, induced by solid state diffusionless, reversible phase transformation between austenite, the high temperature phase, and martensite, the low temperature one [1,2]. Two main properties of NiTi, such as superior corrosion resistance and super long fatigue life, make this material suitable for smart engineering structures and medical applications. Nevertheless, NiTi is extremely difficult to process by conventional processes [3]. Casting problems, such as segregation of alloying elements and the rapid work hardening and superelasticity of NiTi, make conventional machining a challenge and leads to poor quality workpieces. Although new processing approaches, particularly for NiTi machining, have been proposed [4], powder metallurgy (PM) has been demonstrating its efficiency, particularly in what concerns additive manufacturing (AM). Direct processes, such as selective laser melting (SLM), creates a material with homogeneous microstructure and stable properties [5]. Current research on the AM of NiTi parts from prealloyed powders has been associated with difficulties concerning chemical homogeneity and chemical composition control caused by Ni evaporation during the melting process [6]. Moreover, the limits of SLM are mainly related to the ability to achieve complex microshapes and internal microfeatures, as well as high dimensional accuracy. These limitations open new paths to the indirect additive process (shaping = extrusion of a filament, debinding, and sintering) and denominated material extrusion (MEX) [7]. Research studies on NiTi and other metallic powder show the viability and importance of this AM technology to process 3D objects [8,9]. Nevertheless, MEX has some geometrical limitations of PIM (Powder Injection Moulding).

The main target of this study is to attain a suitable NiTi-based material with high densification and appropriate microstructure after MEX, compatible with a crack’s sensor and predictive of component/system failure. Prealloyed NiTi powder is pointed out as the best solution for additive processes. A uniform austenitic structure, a suitable composition, and transformation temperatures for stress-induced martensitic transformation must be its characteristics. Nevertheless, secondary phases originated during prealloyed powder production (atomization) oblige us to search for mechanisms that could contribute to decreasing the Ni in excess by producing NiTi from NiTi_2,_ mainly by avoiding the formation of Ni_3_Ti during processing [8]. Adding TiH_2_ in different percentages can contribute to this target, particularly by favoring the disappearance of Ni_3_Ti. In addition, using TiH_2_ instead of Ti can protect powder from oxidation during post- shaping heat treatments (debinding and sintering) and create “brown” inside, a reducing atmosphere (TiH_2_ decomposition temperature is lower than the temperature of post-heat treatments defined for NiTi [10]). The main strategy to reduce oxidation of Ti is to use TiH_2_. Moreover, TiH_2_ dehydrogenation releases Ti that could react with Ni and promote the formation of NiTi [11]. Dehydrogenation occurs up to 600 °C [12] to 650 °C [11,13], or 700 °C [10,14]. Although there is no defined temperature for dehydrogenation, all the temperatures mentioned are lower than the sintering temperature (1165 °C). Thus, total dehydrogenation is expected before sintering. Another advantage of using TiH_2_ is that dehydrogenation will expose activated Ti enhancing the sintering process, meaning higher density; the oxygen and nitrogen pickup is expected to be lower [15,16]. However, some authors state that when using TiH_2_, pore size reduces, but with more occurrence and is consistently distributed. Different authors studied the effect of TiH_2_ addition with Ni elemental powders to obtain NiTi (Table 1). Li et al., in 1998 and 2000, observed in conventional powder processing that when using TiH_2_, the general porosity and open-pore ratio tend to decrease, pore size also decreases, and the number of pores increases and becomes more uniform, meaning a reduction in shrinkage [17,18]. When increasing TiH_2_ vol.%, together with temperature, sintering is enhanced, contributing to the formation of Kirkendall pores and the shrinkage of the large ones, which is also associated with the enhancement of the shape memory effect (SME). The phases present were NiTi, NiTi_2_, and Ni_3_Ti [17,18]. Bertheville et al. showed the presence of NiTi (B2), Ni_4_Ti_3_, Ni_3_Ti, Ni_2_Ti_4_O_x_, and TiC_0.7_N_0.3_ in the unpolished surface characterization [19]. The two last ones result from contamination of the virgin powder particles during processing [19]. After post-processing, the most prominent phases were NiTi (B2), Ni_4_Ti_3_, and Ni_2_Ti_4_O_x_ [19]. Chen et al. used elemental compositions of 51 at.% of Ni with TiH_2_ and observed a reduction in pore size and an increase in their number associated with a uniform distribution [11]. One significant difference was that Ni-Ti 3D objects swelled and Ni-TiH_2_ shrinkage was observed. The most prominent phase was NiTi (B2), and the occurrence of NiTi_2_ and Ni_3_Ti was reduced compared with Ni-Ti virgin powder [20]. Bohua et al. observed that after sintering with Ni-Ti powder, among the NiTi main phase, NiTi_2_, Ni_4_Ti_3_, TiO_2_, and TiC phases were detected [12]. However, when using Ni-TiH_2_, the TiO_2_ and TiC phases were absent due to the reducing environment formed by the released hydrogen after dehydrogenation. When using Ni-TiH_2_ powder, the 3D object presented a much smaller mean pore size and a homogeneous pore distribution [12].

Studies with TiH_2_ and Ni elemental powder particles used to tune prealloyed NiTi shaped by an additive process were not yet carried out in-depth. The use of TiH_2_ could solve some problems encountered when processing NiTi from prealloyed powder, mainly by promoting sintering kinetics and hindering the formation of pernicious secondary phases. Hydrogen, as a reducing atmosphere, can promote good performance outside and inside the 3D objects. During cooling, the remaining H_2_ should reconnect to Ti, preventing the formation of secondary phases such as Ni_2_Ti_4_O_x_. The disadvantage of this mechanism is that it could lead to the formation of NiTi_2_ due to the presence of free Ti. However, as referred, this phase can contribute to high densification in post-treatments. In addition, it is also important to highlight that studies where no binder is used could be the explanation for the low presence of oxides and carbides. However, in MEX, the presence of organic materials (binder and additives) constitutes a challenge that must be overcome.

## 2. Materials and Methods

**Figure 1:**
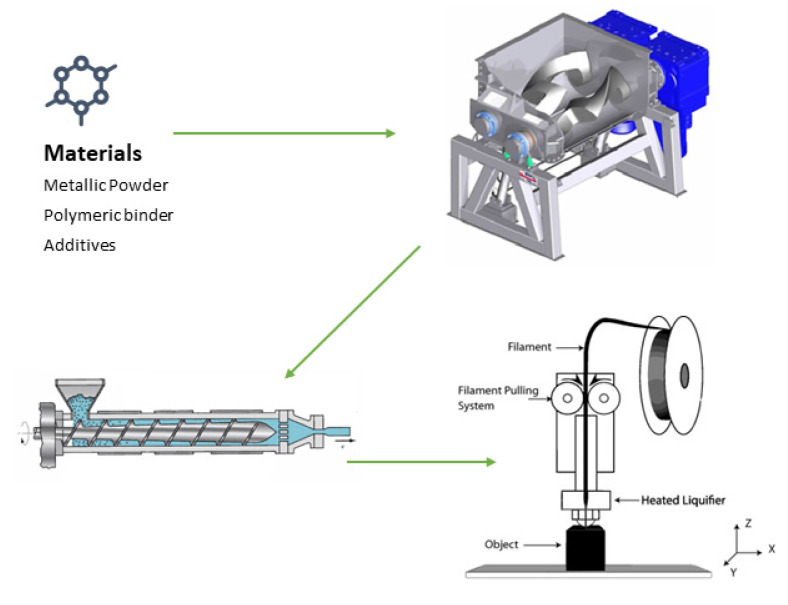
Flowchart of the MEX process (adapted from [21,22]).

The flowchart of the MEX process starting with the mixture of the NiTi powder with binder and additives is shown in Figure 1.

Prealloyed powder is the elective powder for SLM because the elemental Ni and Ti powder is predisposed to form NiTi_2_ and Ni_3_Ti intermetallics due to its high contamination by N_2_ and O_2_. Thus, the option for MEX was also prealloyed powder, with the expectation to yield the main targets of the SLM process, in particular to attain maximum densification and a more uniform microstructure. The virgin prealloyed NiTi powder particles were supplied by LPW Technology Ltd. (Runcorn, UK), nickel powder particles by Sandvik (Sandviken, Sweeden), and TiH_2_ powder particles by Reade Advanced Materials (Riverside, RI, USA). Particle size distribution (PSD) was evaluated using laser diffraction spectrometry LDS, Malvern Panalytical (Egham, UK) with a Malvern Mastersizer 3000. A Philips X’Pert diffractometer (Egham, UK) at 40 kV with Bragg–Brentano geometry (θ–2θ), with cobalt anticathode (λ(kα1) = 0.178897 nm and λ(kα2) = 0.179285 nm), and a current intensity of 35 mA was used to perform phase analysis. The x-ray diffraction scans were carried out from 20 to 100° in steps of 0.025°, with an acquisition time of 1 s per step.

**Figure 2:**
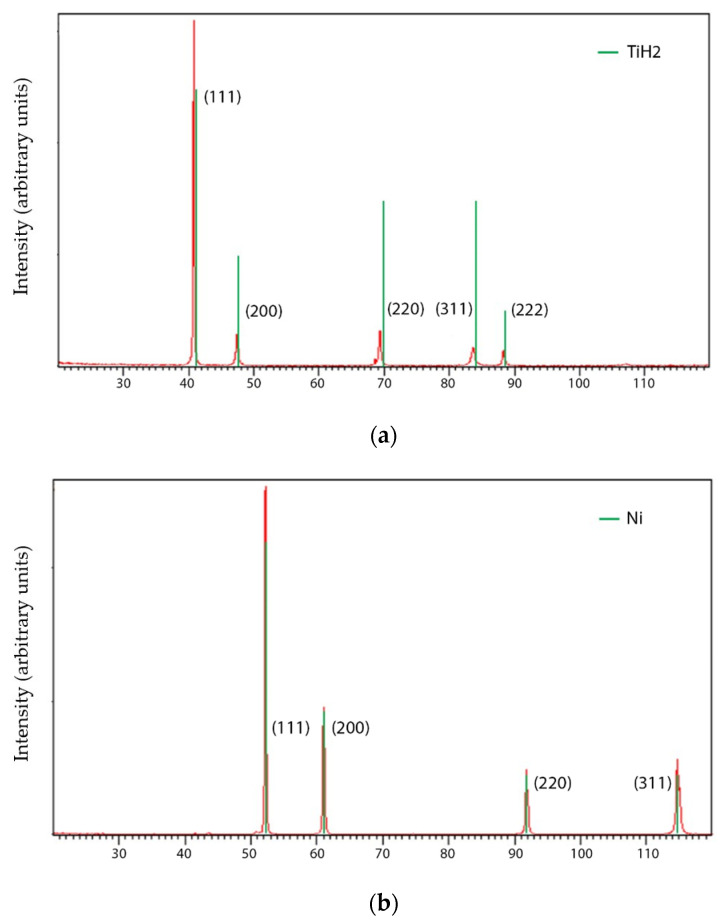
X-ray diffractograms of (**a**) TiH_2_ and (**b**) Ni powder.

Characteristics of NiTi prealloyed powder, binder, and additives are described elsewhere [8]. Phase analysis by X-ray diffraction (XRD) of the prealloyed powder revealed a phase other than NiTi and Ni; it also included NiTi_2_ [8]. TiH_2_ and Ni powder particles have a unique phase present (Figure 2). Particle size analysis shows distinct sizes of the different powder particles. This multiplicity of particle sizes can be a promotor of density during the sintering process [23] (Table 2). Moreover, the D50 of powder particles is not the ideal where sintering is the consolidation step. In MEX, to guarantee an effective solid diffusion among powder particles, D50 should be lower than 10 µm.

The evaluation of the critical powder volume concentration (CPVC) [24,25,26] methodology used in powder injection molding (PIM) feedstocks allows for the optimization of the NiTi filament composition (NiTi powder, master binder, and additives). A torque rheometer, Plastograph Brabender GmbH and Co. (Duisburg, Germany) with a rotation blade speed of 30 rpm at a temperature of 180 °C, was used to optimize the feedstock. The feedstock was granulated and the filament shaped using a single screw extruder Brabender GMBH & Co. E 19/25 (Duisburg, Germany) without a calibration system and with a nozzle diameter of 1.75 mm. The temperatures in different zones of the extrusion cylinder were 170, 175, and 180 °C (nozzle). In order to confirm the quality of the filament for the additive process (MEX) and function of the powder mixture, several mechanical tests were performed. The equipment was a Stable MicroSystems (Godalming, UK). Specimens with 25 mm in length, randomly removed from the filament spool, and were characterized by tensile and three-point bending tests with a 5 kN loading cell; tensile tests were carried out with a loading rate of 0.5 mm min^−1^ and a gauge length of 10 mm; for the three-point bending tests, the span size was 20 mm. For both tests (tensile and bending), twenty specimens of filament (green) were tested at room temperature for each reference powder particle:

A. NiTi prealloyed powder;
B. NiTi prealloyed powder + 1 wt.% TiH_2_;
C. NiTi prealloyed powder + 5 wt.% TiH_2_;
D. NiTi prealloyed powder + 5 wt.% TiH_2_ and 6.2 wt.% Ni.

Solidworks software from Dassault Systèmes [27] was used to create the 3D models and to export the STL file. The G-Code was created with CURA software from Ultimaker B.V. [28]. A Hephestos2 from BQ (Madrid, Spain) with a nozzle diameter of 0.4 mm was used to create the 3D objects.

The thermal consolidation of the “green” filament/3D object was performed in two steps (debinding followed by sintering) in an H_2_ atmosphere. The dwelling times and temperatures were previously optimized [8]. Debinding was performed at a heating rate of 10 °C min^−1^ up to 600 °C followed by sintering at a heating and cooling rate of 5 °C min^−1^ up to 1165 °C during 5 h in a MIM3002T furnace ELNIK Systems (Cedar Grove, NJ, USA). Optical microscopy (OM) and scanning electron microscopy (SEM) FEI Quanta 400 FEG ESEM/EDAX Genesis, Thermo Fisher Scientific (Waltham, MA, USA) were used to analyze the 3D objects. Thermal analyses of sintered parts were performed by differential scanning calorimetry (DSC), allowing for the transformation temperatures to be evaluated. The DSC analysis were carried out in a DSC 204 F1 Phoenix equipment (NETZSCH-Gerätebau GmbH, Selb, Germany), with thermal cycles from −150 °C to + 150 °C and a heating/cooling rate of 10 K.min^−1^. Hardness was evaluated by microhardness testing with HMV equipment from Shimadzu (Kyoto, Japan). Four specimens of each composition were measured 40 times using a maximum load of 10 g. Surface and inside defects of filaments and strands were evaluated by X-ray microcomputed tomography using a Bruker SkyScan 1275 (Bruker, Kontich, Belgium). An acceleration voltage of 80 kV and a beam current of 125 μA was set while using a 1 mm aluminum filter with step-and-shoot mode. Pixel size was set to 6 μm and random mode was used. The images were acquired at 0.2° angular step with five frames average per step using an exposure time of 46 ms. The microCT images were reconstructed with the dedicated manufacturer software.

## 3. Results and Discussion

A steady state must occur to ensure homogeneity in the mixtures, which is crucial to prevent the formation of secondary phases where the ratio of Ni:Ti is unbalanced. The values of torque for A, B, C, and D are quite similar. However, there is a tendency for a slight increase of torque with the increase of TiH_2_ and/or Ni (Table 3). Filaments for all compositions were produced with a CPCV of 60 vol.% of powder particles content, which was the best compromise with the torque value.

**Figure 3:**
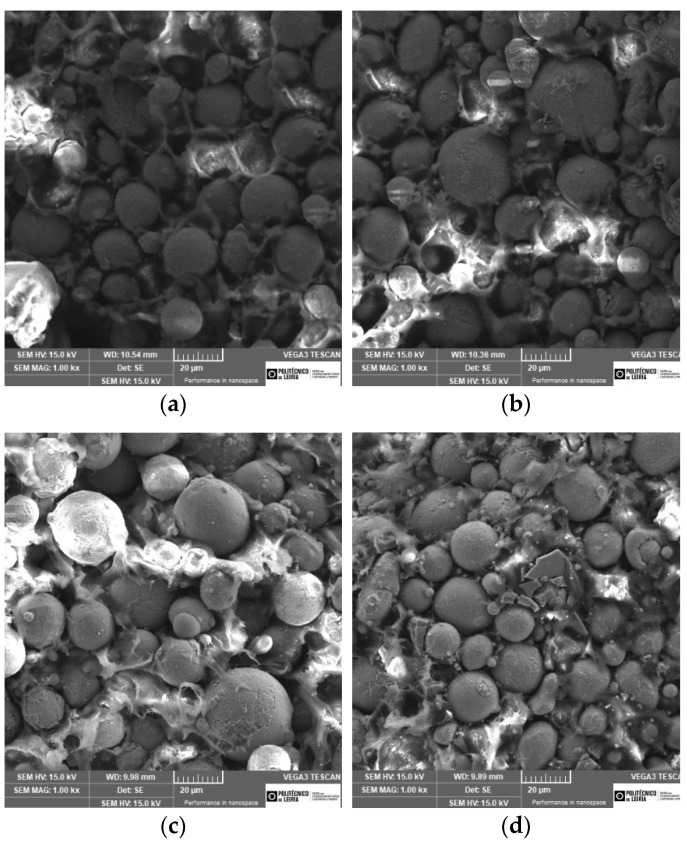
Micrographies of the green filaments (SEM), (**a**) A, (**b**) B, (**c**) C, and (**d**) D.

Figure 3 shows microstructures of the green filament cross sections where a multitude of sizes from their constituents is visible. All filaments show similitudes, with a good distribution of the multiple particle sizes, which is good to attain an excellent interparticle closeness. This is very important, keeping in mind that the powder particles suitable to indirect additive process must have D50 lower than 10 µm.

In filaments A, B, and C, the particles have a shape factor close to 1. However, in filament D (Ni addition), some sharpened particles are observed.

Regarding the mechanical properties, the Young modulus values are very similar for all compositions (Table 4). The filaments reveal a similar behavior on elastic domain, whatever the feedstock selected.

Three-point bending tests were performed to highlight the filaments homogeneity/reproducibility by the Weibull index (m). This index, when greater than 10, is an indicator of reproducibility of the green filament. The Weibull modulus from the 3-point bending test show significative difference between filaments A, B, and C to the filament D, which has a value almost the double of the other ones (Table 5). This behavior can be attributed to the multiplicity of particle sizes of the different added powder and excellent homogeneity.

The shaping, debinding and sintering (SDS) were previously optimized, and the conditions of processing for all compositions are described elsewhere [8]. Sintering of the prealloyed powder (1165 °C) must be enough to guarantee the consolidation of the powder particles, without formation of other intermetallic phases, different from the existent in virgin powder (NiTi + NiTi_2_ + Ni) [8]. The sintering temperature (1165 °C) is enough to melt the NiTi_2_ phase (Tm = 984 °C), which can contribute to a liquid phase sintering, accelerating the densification and homogenization processes.

**Figure 4:**
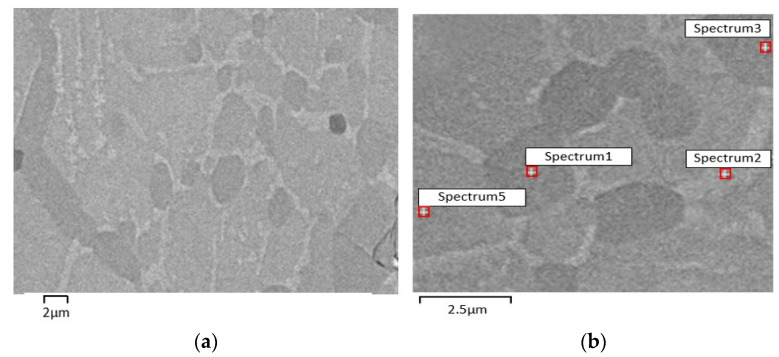
Micrographies of 3D object from filament A. (**a**) after sintering (SEM), (**b**) selected zones for evaluation of Ni:Ti ratio (S1, S2, S3 and S5) by SEM/EDS.

**Figure 5:**
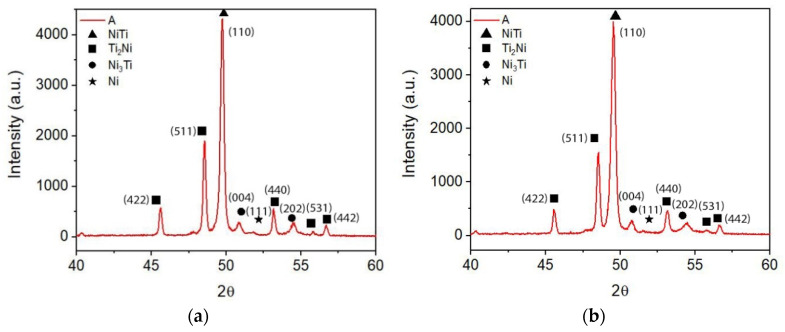
X-ray diffractograms of 3D object from filament A. (**a**) top, (**b**) bottom.

After sintering, the SEM micrographies (backscattered electrons, BSE) suggest the appearance of a new phase (S2) rich in Ni (Ni_3_Ti) (Figure 4, Table 6). X-ray diffraction of sintered A (standard) shows: NiTi as the master phase, NiTi_2_ already present in virgin powder, Ni_3_Ti resulting from the diffusion of loose nickel into NiTi and NiTi_2_ and residual Ni (Figure 5). The semi-quantitative analysis of A shows a significant difference between NiTi and NiTi_2_ volume percentages (85:15). The white phase distributed around the different grains of NiTi can be attributed to Ni_3_Ti (Figure 4).

**Figure 6:**
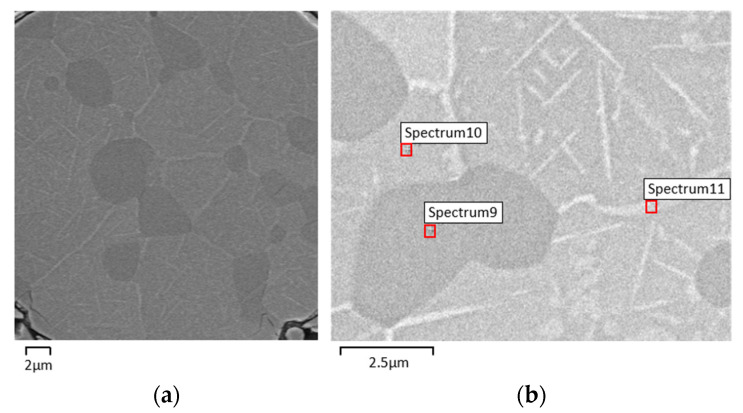
Micrographies of 3D object from filament B (addition of 1 wt.% TiH_2_). (**a**) after sintering (SEM), (**b**) selected zones for evaluation of Ni:Ti ratio (S9, S10, and S11) by SEM/EDS.

**Figure 7:**
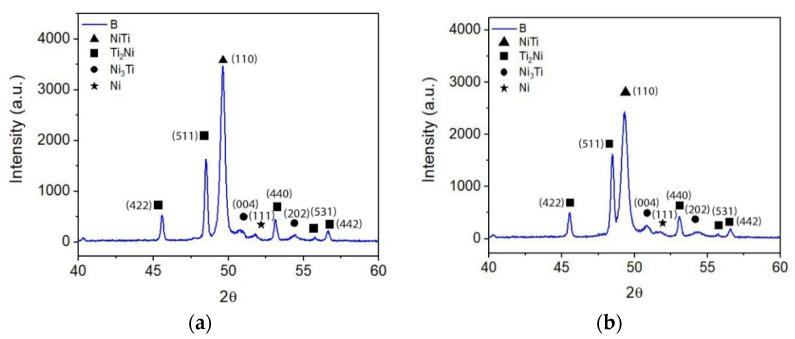
X-ray diffractograms of 3D object from filament B. (**a**) top, (**b**) bottom.

With the addition of 1 wt.% of TiH_2_ to NiTi prealloyed powder, no notorious difference is observed. Based on the colors of the SEM micrographies (BSE) and EDS results, three distinct phases (NiTi, NiTi_2,_ and Ni_3_Ti) are identified (Figure 6, Table 7). The x-ray diffractogram analysis clearly shows the presence of NiTi, NiTi_2,_ and Ni_3_Ti and also Ni from virgin powder (Figure 7). In what concerns the percentages of NiTi and NiTi_2,_ there is a tendency for a small increase of NiTi_2_ percentage in filament B.

**Figure 8:**
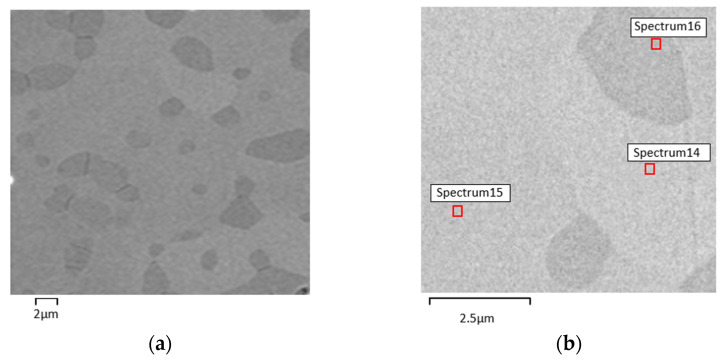
Micrographies of 3D object from filament C (addition of 5 wt.% TiH_2_). (**a**) after sintering (SEM), (**b**) selected zones for evaluation of Ni:Ti ratio (S14, S15 and S16) by SEM/EDS.

The micrographies of 3D objects from composition C (NiTi + 5 wt.% TiH_2_) show a significant difference from the other compositions. The white phase, identified as Ni_3_Ti, is not present in composition C. Similar to the other compositions, the Ni:Ti ratio also suggests the formation of phases constituted by Ni and Ti, although enriched in Ni, such as Ni_3_Ti_2_ and/or Ni_4_Ti_3_ [29,30,31,32] (Figure 8, Table 8). Moreover, a slight increase of the NiTi_2_ content is also evident.

**Figure 9:**
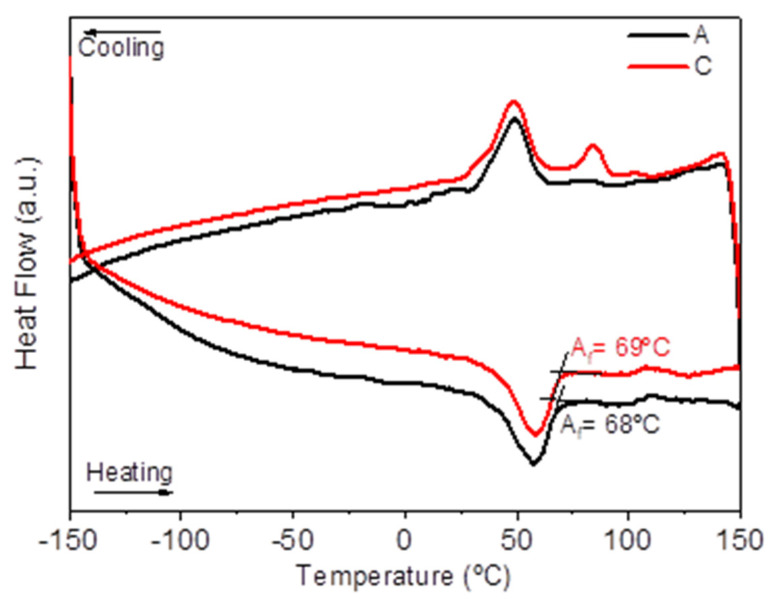
DSC curves of 3D objects from filaments A and C (addition of 5 wt.% TiH_2_).

The DSC curves in Figure 9 show the influence of 5 wt.% TiH_2_ addition (3D object from filament C) to NiTi (3D object from filament A). The phase transformation temperatures are above room temperature for both cases, which might indicate a Ti-rich NiTi matrix [2]. The final austenite phase transformation temperature slightly increased with the TiH_2_ addition (A_f_ (A) = 68°C and A_f_ (C) = 69°C). Moreover, a 3D object from filament C displays the presence of R-phase on cooling, probably due to the increase of the Ti content.

**Figure 10:**
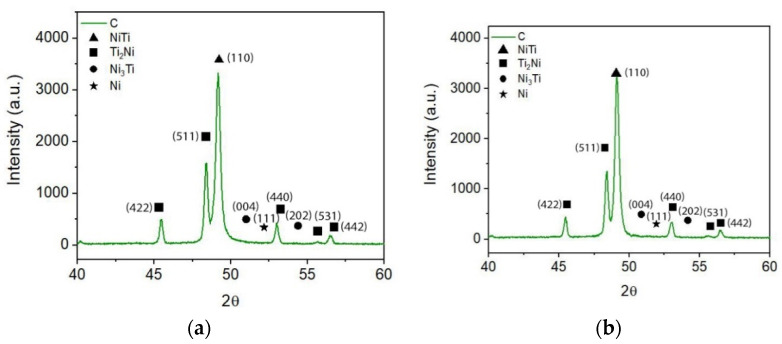
X-ray diffractograms of 3D object from filament C. (**a**) top, (**b**) bottom.

X-ray diffractograms corroborate the SEM results in the apparent disappearance of the Ni_3_Ti phase. Moreover, they suggest the possibility that Ti, resulting from dehydrogenation, may have contributed to the formation of NiTi. The XRD results also support the possible reaction of free Ti resulting from dehydrogenation with free Ni present in the virgin powder, since Ni is not identified in the x-ray diffractograms (Figure 10).

**Figure 11:**
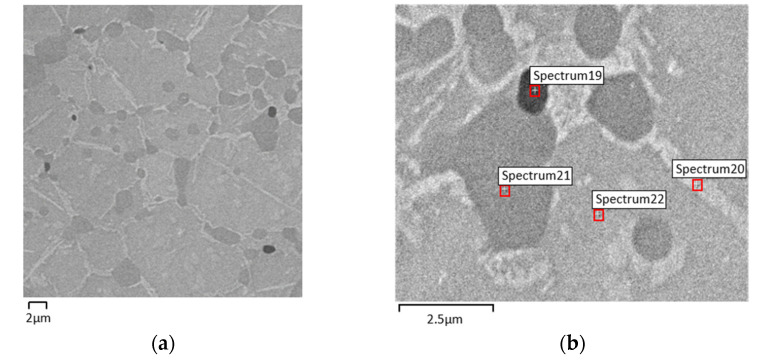
Micrographies of 3D objects from filament D (addition of 5 wt.% TiH_2_ and Ni). (**a**) after sintering (SEM), (**b**) selected zones for evaluation of Ni:Ti ratio (S19, S20, S21 and S22) by SEM/EDS.

Composition D has a supplementary content of Ni (6.2 wt.%) mixed with virgin powder (NiTi + NiTi_2_ + Ni) and with 5 wt.% TiH_2_. This composition has two objectives: first to highlight the role of the excess of Ni in the Ni_3_Ti phase formed during processing, and the second, to analyze the role of the excess of Ni in the disappearance of NiTi_2_ resulting from NiTi powder fabrication. In fact, with the addition of Ni, a drastic decrease of the NiTi_2_ is observed, as evidenced in the SEM images of 3D object D (Figure 11) when compared to B (Figure 6) and C (Figure 8) 3D objects. Thus, powder Ni content could be tuned as a possible solution for the disappearance of NiTi_2_ in order obtain only NiTi in prealloyed powders.

**Figure 12:**
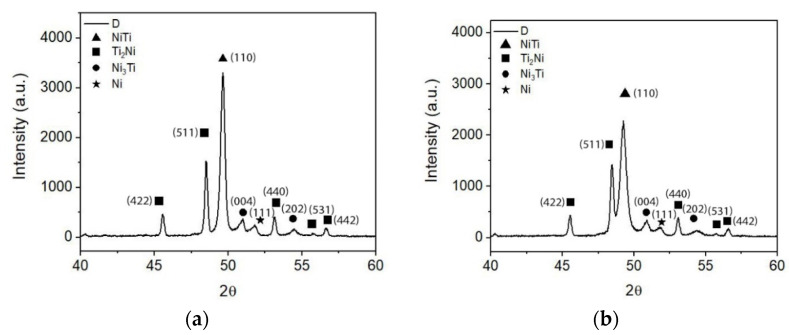
X-ray diffractograms of 3D object from filament D (**a**) top, (**b**) bottom.

Similar to sintered 3D objects from filaments with TiH_2_ lower than 5 wt.%, micrographies and x-ray diffractograms from 3D objects with composition D (Ni in excess, other than the pristine one) show again the formation of a white phase identified as Ni_3_Ti. Despite the addition of Ni, Figure 11 and Table 9 show the occurrence of a “new phase” almost depleted of Ni, suggesting the presence of Ti without any reaction with other metal present. However, there are no discernible Ti peaks in the x-ray diffractogram (Figure 12).

Considering that during sintering the 3D objects are on a platform that could compromise the process and originate the formation of new phases, both the top and base were analyzed by XRD. It is clear that the top and base of 3D objects show similar phase composition, meaning that all binder and additives were effectively removed, and the sintered phases are similar.

**Figure 13:**
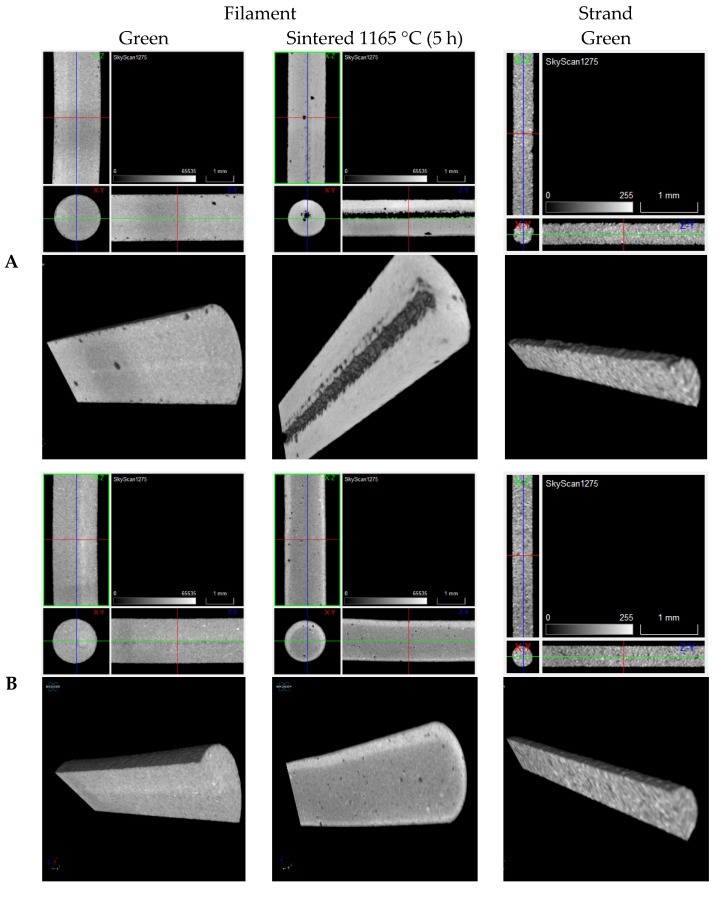

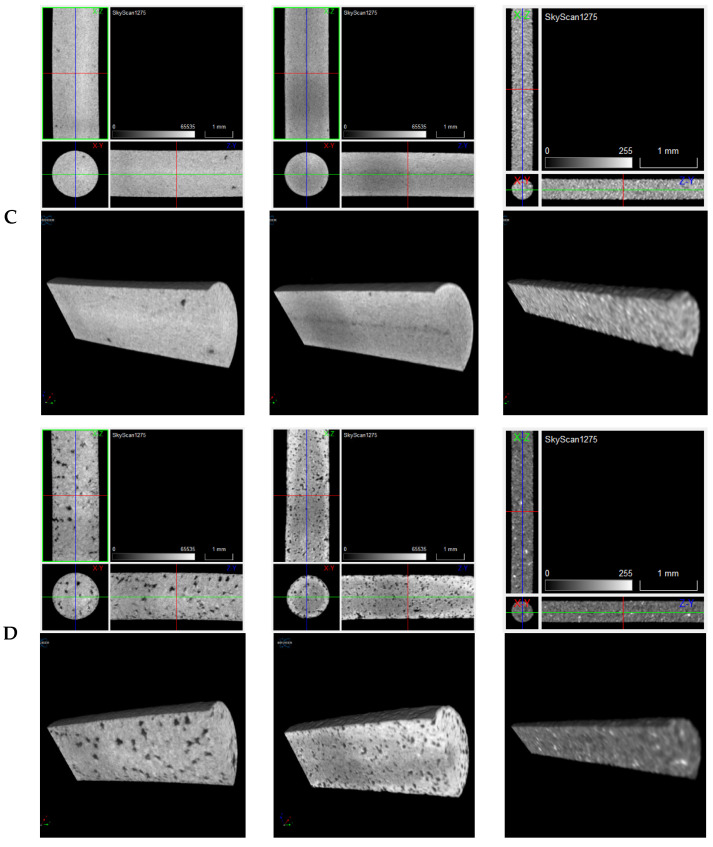
Tomographies of (**A**–**D**) filaments (green and sintered at 1165 °C during 5 h) and strands (**A**–**D**) (green).

Tomography analysis is of enormous importance to detect failures inside the green and sintered 3D objects. For some compositions, detailed analysis of filaments defects before and after debinding and sintering reveals a significant presence of porosity, inside and at the surface. The defects are mainly present in filament D. Filaments A, B, and C, sintered at 1165 °C for 5 h, show a low quantity of defects against D that shows a significant content of porosity (Figure 13). Defects in the strands can be inherited from filaments and consequently transmitted to the 3D object. A relation can be observed between filaments and strands of composition D that also shows a large amount of porosity and surface defects (Figure 13).

**Figure 14:**
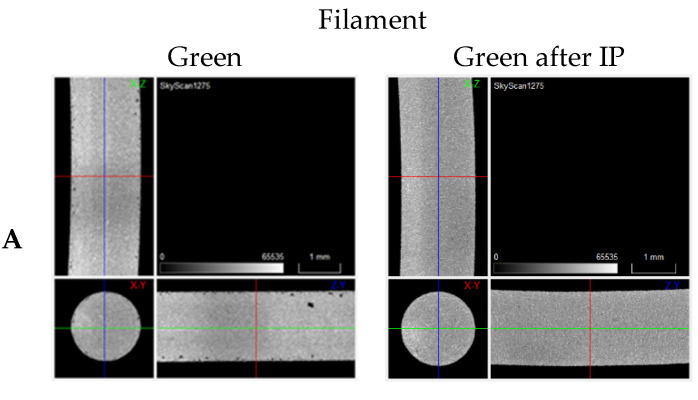

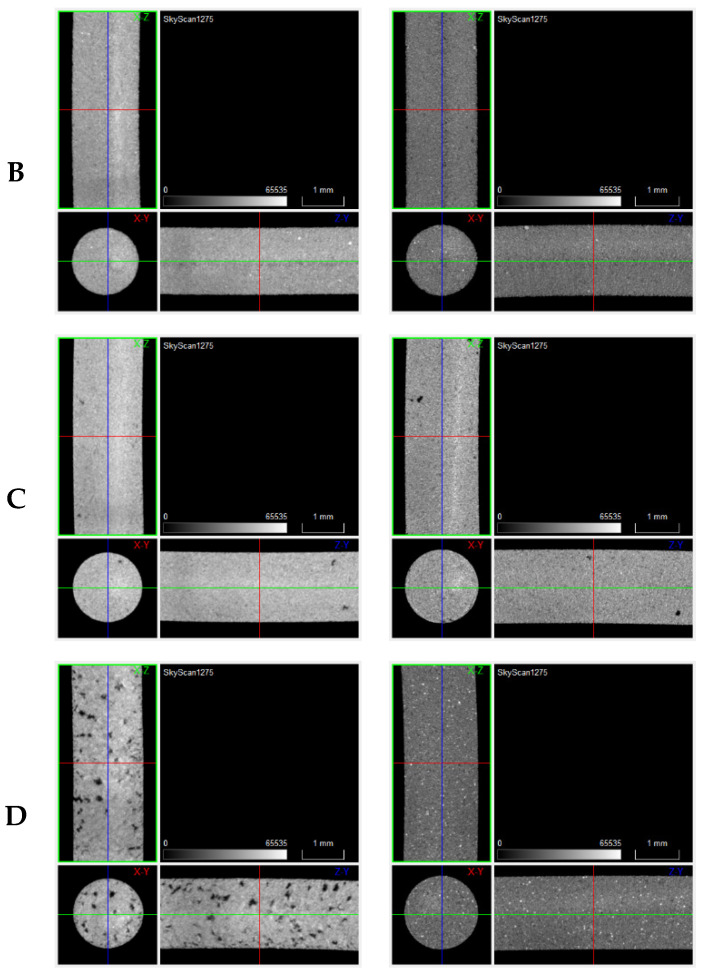
Tomographies of green vs. IP green filaments (**A**–**D**).

As a complement, the study of isostatic pressing (IP) was performed in the green filaments. IP is one of the most significant treatments to decrease porosity in filaments/3D objects in the green state (Figure 14). As expected, the most relevant observation is the reduction of porosity in Filament D.

Indirect additive manufacturing, such as MEX, could be the sustainable technology ideal for applications where the geometry envisaged could be complex, but the thickness is less than 3 to 5 mm. Moreover, the densification could be improved by the formation of a liquid phase during sintering, allowing the sintering temperature/time to be decreased. For densification, the mechanism of LPS is valid in a system with a very small volume fraction of liquid (e.g., NiTi_2_), so that the liquid is present only in the neck region between particles. The pore filling mechanism is justified for LPS, where the grain maintains an equilibrium shape. The microstructural evolution observed in the system studied supports the pore filling [33].

Hardness values are higher than the hardness of bulk NiTi (NiTi (B2) 275 HV, NiTi (B19) 112 HV, NiTi_2_ 163 HV and Ni_3_Ti 1071 HV [34,35,36,37]), confirming the presence of hard phases (i.e., Ni_3_Ti) (Table 10). The hardness values are similar to those of NiTi 3D objects obtained from other non-conventional technologies (800 HV [38], 700 HV [39,40], and 742 HV [41]).

In composition C where Ni_3_Ti was not detected, a lower hardness was expected. Instead, composition D, where Ni_3_Ti was detected, presents the lowest value. A possible explanation for the decrease in hardness observed for composition D is the presence of the Ti-phase previously identified.

## 4. Conclusions

NiTi SMA 3D objects manufactured from prealloyed powder by MEX with the lowest possible porosity with a uniform and suitable microstructure were the main objective of the present study.

The presence of NiTi_2_ with low melting temperature (984 °C) and Ni in the prealloyed powder are expected outcomes of the atomization process. The NiTi_2_ phase can convert the conventional consolidation process of NiTi based on solid diffusion in a liquid phase sintering process. In addition to decreasing the porosity, the NiTi_2_ intermetallic phase can also have a significant role when sintering is the consolidation process because it can contribute to the uniformization of the final microstructure. The porosity can be significantly reduced by the isostatic pressing of greens (P = 100 GPa, time = 2 h).

Both NiTi_2_ and free Ni would be suitable to promote NiTi formation during the liquid phase sintering. The addition of 5 wt.% of TiH_2_ to virgin prealloyed powder highlights that Ti (released after dehydrogenation), together with free Ni from pristine powder, contributes to the formation of NiTi instead of Ni_3_Ti and total depletion of the loose Ni. The composition of prealloyed powder with 5 wt.% TiH_2_ showed after sintering a homogeneous matrix, but yet with a NiTi_2_ second phase uniformly dispersed. The sintering process was excellent and for all the mixtures studied the phases formed, both at the top and bottom, were similar.

Therefore, the use of MEX for processing NiTi prealloyed powder particles showed promising results, opening a field to new applications of NiTi, namely as a sensor. In the future, the role of NiTi_2_ in the detection of failure cracks by mechanical sensors must be demonstrated.

## Figures and Tables

**Table 1:** Sintering and post-processing conditions of elemental Ni-TiH_2_ powder and final phases.

Temperature [°C]	Holding Time [h]	Vacuum Pressure [Pa]	Processing Technology	Porosity after Sintering (%)	Post-Processing	Phases	Ref.
950	1	1.33 × 10^−2^	Pressing	33.9–37.6	-	NiTi NiTi_2_/Ni_2_Ti_4_O_x_ Ni_3_Ti	[17,18]
920	14	-	Pressing	29–34	Aged (Ar) 500 °C (1 h) Not polished	NiTi Ni_2_Ti_4_O_x_ Ni_4_Ti_3_ Ni3Ti TiC0.7 N0.3	[19]
					HIP (Ar) 180 MPa 1050 °C (3 h) Aged (Ar) 500 °C (1 h) Not polished	NiTi Ni_2_Ti_4_O_x_ Ni_3_Ti TiC_0.7_ N_0.3_	
					Aged (Ar) 500 °C (1 h) Polished	NiTi Ni_2_Ti_4_O_x_ Ni_4_Ti_3_	
					HIP (Ar) 180 MPa 1050 °C (3 h) Aged (Ar) 500 °C (1 h) Polished		
					HIP (Ar) 180 MPa 1050 °C (3 h) Annealed (Ar) 1100 °C (1 h) Aged (Ar) 500 °C (1 h) Polished		
1000	2	3 × 10^−3^	Pressing	10–33.8	-	NiTi NiTi_2_ Ni_3_Ti	[11]
1100							
1200							
900	2	3 × 10^−3^	Pressing	19 (>900 °C)	-	Ni Ti NiTi NiTi_2_ Ni_3_Ti	[20]
950							
1000							
1100							
1200							
1100					1000 °C 6 h vacuum	NiTi NiTi_2_	
1200							
1000	2	1 × 10^−3^	Gel Casting	40–46	-	NiTi NiTi_2_ Ni_4_Ti_3_	[12]

**Table 2:** NiTi, TiH_2,_ and Ni powder particles size, particle size distribution, and specific surface area (SSA).

	NiTi	TiH_2_	Ni
D_10_ [µm]	13.4	3.4	21.4
D_50_ [µm]	22.1	15.3	30.0
D_90_ [µm]	34.7	35.7	41.2
SSA [m^2^ kg^−1^]	293.4	750.2	207.7

**Table 3:** Steady state torque.

	A	B	C	D
Steady state torque [Nm]	4.2	4.0	4.6	4.8

**Table 4:** Young modulus of the green filaments (powder + binder + additives).

	A	B	C	D
Young modulus [GPa]	2.6 ± 0.11	2.8 ± 0.15	2.7 ± 0.14	2.5 ± 0.28

**Table 5:** Weibull modulus of green filaments from 3-point bending tests.

Tests	A	B	C	D
3-point bending	23	29	28	45

**Table 6:** 3D object phases from filament A after sintering (Spectra(S) 1, 2, 3, and 5 in Figure 4b).

Phase Composition (EDS)			
S1	S2	S3	S5
NiTi_2_	Ni_3_Ti	NiTi_2_	NiTi

**Table 7:** Phases from filament B after sintering (Spectra 9, 10, and 11 in Figure 6b).

Phase Composition (EDS)		
S9	S10	S11
NiTi_2_	NiTi	Ni_3_Ti

**Table 8:** Three-dimensional object phases from filament C after sintering (Spectra 14, 15, and 16 in Figure 8b).

Phase Composition (EDS)		
S14	S15	S16
NiTi	NiTi	NiTi_2_

**Table 9:** Three-dimensional object phases from filament D after sintering (Spectra 19, 20, 21, and 22 in Figure 11b).

Phase Composition (EDS)			
S19	S20	S21	S22
Ti	Ni_3_Ti	NiTi_2_	NiTi

**Table 10:** Hardness of the sintered filaments/3D objects (1165 °C, 5 h).

	A	B	C	D
Hardness [HV_0.01_]	887 ± 58	773 ± 68	715 ± 39	677 ± 59

## Data Availability

Data sharing not applicable to this article.
